# Characterization of the Vitrocell® 24/48 *in vitro* aerosol exposure system using mainstream cigarette smoke

**DOI:** 10.1186/s13065-014-0062-3

**Published:** 2014-11-12

**Authors:** Shoaib Majeed, Stefan Frentzel, Sandra Wagner, Diana Kuehn, Patrice Leroy, Philippe A Guy, Arno Knorr, Julia Hoeng, Manuel C Peitsch

**Affiliations:** Philip Morris Research and Development, Quai Jeanrenaud 5, 2000 Neuchâtel, Switzerland; Eurofins Umwelt West GmbH, Vorgebirgsstraße 20, D-50389 Wesseling, Germany

**Keywords:** Air–liquid interface, Cigarette smoke, Nicotine, Carbonyl, *In vitro* exposure system, Vitrocell®

## Abstract

**Background:**

Only a few exposure systems are presently available that enable cigarette smoke exposure of living cells at the air–liquid interface, of which one of the most versatile is the Vitrocell® system (Vitrocell® Systems GmbH). To assess its performance and optimize the exposure conditions, we characterized a Vitrocell® 24/48 system connected to a 30-port carousel smoking machine. The Vitrocell® 24/48 system allows for simultaneous exposure of 48 cell culture inserts using dilution airflow rates of 0–3.0 L/min and exposes six inserts per dilution. These flow rates represent cigarette smoke concentrations of 7–100%.

**Results:**

By characterizing the exposure inside the Vitrocell® 24/48, we verified that (I) the cigarette smoke aerosol distribution is uniform across all inserts, (II) the utility of Vitrocell® crystal quartz microbalances for determining the online deposition of particle mass on the inserts, and (III) the amount of particles deposited per surface area and the amounts of trapped carbonyls and nicotine were concentration dependent. At a fixed dilution airflow of 0.5 L/min, the results showed a coefficient of variation of 12.2% between inserts of the Vitrocell® 24/48 module, excluding variations caused by different runs. Although nicotine and carbonyl concentrations were linear over the tested dilution range, particle mass deposition increased nonlinearly. The observed effect on cell viability was well-correlated with increasing concentration of cigarette smoke.

**Conclusions:**

Overall, the obtained results highlight the suitability of the Vitrocell® 24/48 system to assess the effect of cigarette smoke on cells under air–liquid interface exposure conditions, which is closely related to the conditions occurring in human airways.

## Background

Cigarette smoke (CS) is a complex heterogeneous mixture of over 4000 compounds, of which at least 250 have known toxicological effects and are associated with various smoking-related diseases, including respiratory and cardiovascular disorders, and cancer [1]. CS is composed of a gas–vapor and particulate phases, which contain different constituents. For example, the gas–vapor phase fraction contains high levels of aldehydes (carbonyls), whereas the particulate phase fraction contains polycyclic aromatic hydrocarbons [2] and tobacco-specific nitrosamines [2,3] among other components.

*In vitro* studies were designed to elucidate the potential adverse effects of individual components, smoke fractions, or whole smoke on exposed cells. Exposure to individual compounds of CS is usually performed in submerged cell culture systems. Furthermore, compound solutions can be easily diluted in culture media, which allows concentration-dependent effects to be determined. The particulate and gas–vapor phase fractions, trapped by either filter or in solution, are also well-suited for *in vitro* studies using submerged cell cultures systems. However, exposure to whole smoke aerosols *in vitro* is technically difficult and only feasible if the cells are located at the air–liquid interface (ALI). Unlike submerged cell cultures seeded on culture plates, cells growing at the ALI are seeded on porous membranes on culture inserts and are supplied with medium from their basal side, while their apical side is in contact with air. This cell culture system allows exposure to whole smoke, bringing the cells in direct contact with the gas–vapor as well as the particulate phase. Cell lines that were generally used for *in vitro* studies of cigarette smoke include A549 [4], BEAS-2B [5] or 16HBE [6] (at the ALI or as submerged cultures). A549 cells were initially grown from a human alveolar cell carcinoma. Their morphology resembles that of type II alveolar epithelial cells of the lung, and they produce lecithin with a high proportion of saturated fatty acids, similar to pulmonary surfactant [4]. Ke et al. [5] developed a cell line based on human bronchial epithelial cells, which were transfected with the SV40 virus T antigen genes and therefore have an extended culture lifespan. Although cell lines have the advantage that they can be expanded and grow faster to monolayers, three-dimensional (3D)-organotypic cell culture models better mimic the normal structure of the bronchial epithelium with a large ALI, which includes a pseudo-stratified phenotype containing ciliated and non-ciliated cells [7]. However, the production of (3D)-organotypic cell cultures is not straight forward and takes considerable more time. For this reason and since epithelial cell lines can be also grown at ALI condition, we focused in the following work on A549 or BEAS-2B cells, in order to characterize smoke-dependent effects on cell viability.

Although the particulate and gas–vapor phases (or CS condensate) are widely used for *in vitro* studies, they have some important limitations. In particular, the method used for trapping them might alter the chemical composition of each fraction. Some compounds (especially volatile compounds) cannot be trapped quantitatively; the filters or other components of the collection system might leach impurities into the collected material and the solvents used might react with constituents of the smoke fraction. Most importantly, analysis of the individual fractions might underestimate the overall risk attributable to CS through combined exposure to multiple toxicants. For these reasons, researchers are increasingly using culture systems where cells are exposed to CS at the ALI [7-9]. Culture inserts, in which cells grow at the ALI, are placed in an exposure chamber and CS is guided through the chamber in a highly controlled manner using a cigarette smoking machine. Several research groups and companies have developed whole smoke exposure systems that enable *in vitro* exposures of the ALI and submerged cultures. These systems consist of a smoking machine (i.e. Borgwaldt RM20S smoking machine [10], Burghart MSB-01 Mimic Smoker [11,12], and Vitrocell VC 10 smoking robot [13,14]) and cell culture exposure chambers (i.e. Curbridge Engineering/British American Tobacco [14], Cultex® exposure module, Cultex® radial flow system [6,15], and Vitrocell® modules [16]). Although the overall design and specifications of these systems differ slightly (e.g., the basic principle of the Cultex® and Vitrocell® ALI exposure modules was first described by Aufderheide et al. 2001 [17] and later refined by each company), they all allow the researcher to control the concentration of whole smoke.

**Figure 1:**
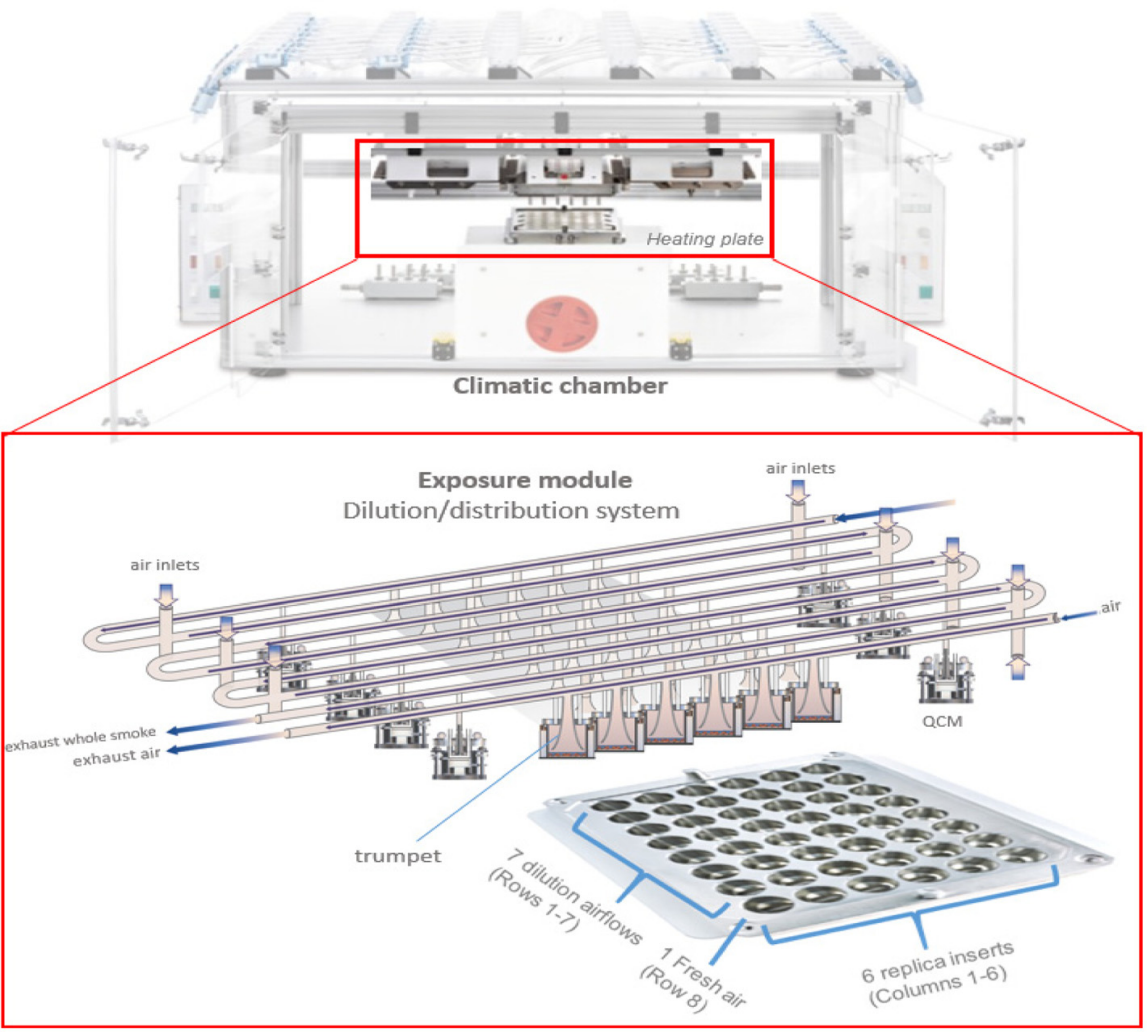
**Schematic representation of the Vitrocell® 24/48 exposure system.** A climatic chamber houses an exposure module that consists of a dilution/distribution system located on top of a cultivation base module. In the base module, up to 48 wells can be simultaneously exposed. The base module has a format of 8 rows × 6 columns (i.e. for 7 dilution airflows and 1 fresh air control row). The delivery of whole smoke is achieved by individual trumpets that deliver the smoke from the dilution/distribution system to the wells of the cultivation base module at a flow rate of 2 mL/min. QCMs are connected to each row of the dilution/distribution system and monitor particle deposition.

The Vitrocell® 24/48 exposure system used in this study has the following features (Figure 1). It is supplied with a double inlet dilution/distribution system for up to seven dilution airflows (by turbulent mixing of CS with fresh air, it creates flow rates ranging from 0.1 to 3 L/min) and one fresh air control. Six replicate wells in the cultivation base module can be exposed to each dilution airflow at a time, and single supply of CS to the replicates is accomplished by individual trumpets. The sampling of CS via the trumpets is accomplished by negative pressure applied by a vacuum pump, which is connected to the cultivation base module. The sampling airflows in the Vitrocell® 24/48 can be adjusted from 2 to 5 mL/min. However, in the experiments outlined below, only 2 mL/min was used. For the generation of CS dilution/distribution, a 30-port carousel smoking machine (SM 2000, Philip Morris Intl.) was used, which pumps the aerosol directly into the inlet of the Vitrocell® 24/48 dilution/distribution system. Humidification of CS was performed by a humidification station (Vitrocell® Systems GmbH) connected to the air inlets of the Vitrocell® 24/48. The system also provides an integrated, sensor-controlled heating plate as well as a climatic chamber surrounding the dilution/distribution system and cultivation base module.

It is notable that, although the overall conditions can be controlled in terms of smoke dilution and smoking regimen, it is difficult to measure the actual deposition of CS on culture inserts. Various methods have been used to analyze the concentration of whole smoke inside *in vitro* exposure systems, although they are specific for the gas–vapor or the particulate phase. These methods include the measurement of particle concentration (e.g., by photometric analysis [18]) or determining gases in the gas–vapor phase (e.g., using CO gas analyzers installed in the exposure system [19]). However, none of these methods reveals the aerosol deposition on the culture inserts. The Vitrocell® system provides such a tool as an integral part of its exposure module. Quartz crystal microbalances (QCMs) were originally developed in the 1950s [20] and have been used to measure environmental pollutants. Particles depositing on the quartz crystal surface are detected by a change in its resonance frequency, which is proportional to the deposited mass. The sensitivity of a QCM is related to the intrinsic property of the crystal (in our case 10 ng/cm^2^, according to Vitrocell® product information). QCMs can be connected to individual rows of the dilution/distribution system of the Vitrocell® 24/48, allowing simultaneous exposure of cells. Using a Vitrocell® VC 10 Smoking Robot and a Vitrocell® 6/4 Stainless Steel module, Adamson et al. [13] conducted a number of studies to evaluate and validate the QCM for dosimetric purposes in CS exposure studies. Based on these studies and the convenience of this tool for online process control inside the exposure system, we decided to use QCMs for the characterization of the Vitrocell® 24/48 system. To combine this analysis, we employed an independent method based on chemical reactivity with an indicator dye. It was noticed that WST-1, a commercially available tetrazolium solution used for cell viability tests (Roche Applied Science), is reduced if exposed to CS, presumably because CS contains a high concentration of oxidants in the gas–vapor and particulate(tar) phases [21]. Reduction of WST-1 leads to the formation of colored formazan, which can be read in a plate reader at an optical density (OD) of 430 nm. WST-1 reduction was therefore used as a simple assay to determine the distribution of CS over the cultivation base module of the Vitrocell® 24/48 system, as well as to characterize the concentration-dependency for increasing concentrations of CS.

In addition to the aforementioned methods, we also determined the concentration of certain CS constituents. As part of this analysis, nicotine and a range of carbonyls were determined in the dilution/distribution system of the Vitrocell®. Finally, the effect of CS on cells placed in the Vitrocell® 24/48 system was evaluated using A549 and BEAS-2B cells.

## Results

### Mainstream CS distribution inside the Vitrocell® 24/48 exposure chamber

**Figure 2:**
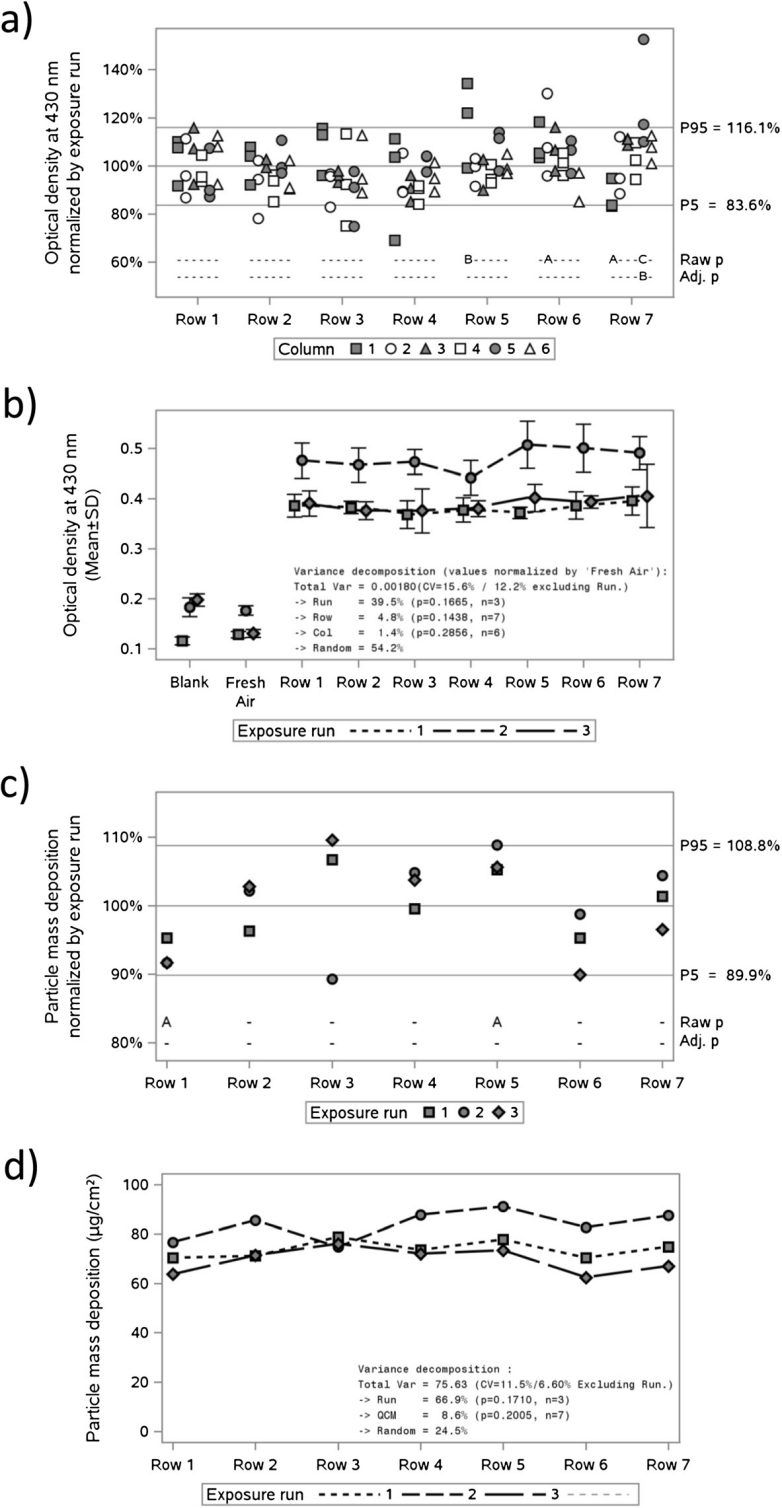
**Uniform distribution (well-to-well variability) of CS aerosols at a single concentration of 32% (0.5 L/min). (a)** Scatter blot showing the blank corrected OD values for WST-1 reduction, normalized to 100%, for each insert of the cultivation base module, from three independent CS exposure runs. Different insert positions in the respective rows of the cultivation base module (Row 1–7) are marked with different symbols. Raw p-values according to ANOM (Raw p) and adjusted p-values for multiple testing (Adj. p) are indicated on the bottom of the graph for each insert position (A: p < 0.05; B: p < 0.01 and C: p < 0.001). **(b)** Average optical density at 430 nm upon WST-1 reduction by CS exposure. Data points represent the mean ± SD from six inserts for CS exposed (rows 1–7) of the cultivation base module, the blank measurement, and exposure to fresh air. Different lines correspond to three different exposure runs, as shown below the graph. **(c)** Scatter blot for particle mass deposition, normalized to 100%, of different QCMs attached to Row 1–7. Raw p-values according to ANOM (Raw p) and adjusted p-values for multiple testing (Adj. p) are indicated on the bottom of the graph for each insert position (A: p < 0.05). Different symbols correspond to three different exposure runs, as shown below the graph. **(d)** The amount of particles measured on QCMs connected to each row of the dilution system (Row 1–7). Different lines correspond to three different exposure runs, as shown below the graph. (a + c) Lines at P95 and P5 refer to the 95 and 5 percentiles, respectively of all values. Statistics based on variance decomposition are shown in each graph. The respective p-values and n-numbers are also listed.

To evaluate the performance of the Vitrocell® 24/48 system, the distribution of CS from reference cigarettes (3R4F) was analyzed using a modified Health Canada smoking protocol [22]. First, the reduction of WST-1 by CS was monitored by measuring its OD at 430 nm after exposure. At the selected dilution airflow of 0.5 L/min, which corresponds to a concentration of 32% CS, an equal distribution of the CS inside the Vitrocell® 24/48 system was revealed. Figure 2a shows blank corrected OD values, normalized to 100%, for each insert in the cultivation base module, from three independent CS exposure runs. Overall, the distribution appeared uniform, exhibiting no regional pattern or gradient. The *p*-values denoted at the bottom of the graph refer to an analysis of means (ANOM) and are descriptively reported as A, B, and C, meaning the *p*-value was below 0.05, 0.01, and 0.001 respectively (Raw p). Given the number of inserts (total 42) and discarding false positives, raw *p*-values were adjusted for multiple testing, which resulted in only a single insert position with significant difference and a *p*-value <0.01 (Adj. p). However, OD values for this particular position contained an extreme data point measured in one of the three exposure runs (i.e. the normalized OD was >140%). The averaged ODs (± standard deviation (SD)) of the six inserts located in each of the rows of the cultivation base module (Figure 2b), showed a clear difference in the exposed rows (rows 1–7) compared to the fresh air exposed control row. Three exposure runs were conducted to estimate their effect on total variability. Runs 1 and 2 were performed on the same day, while run 3 was performed on a different day. Overall, the values from run 2 were slightly higher than those from runs 1 and 3. Variance decomposition analysis confirmed that the main source of variability was the exposure run (39.5% of the total variance). However, the variance decomposition analysis for all CS-exposed inserts indicated a relatively low insert-to-insert variation of a coefficient of variation (CV) of 12.2%, (excluding exposure run effects). The total CV including run-to-run differences was 15.6%. Decomposition analysis also indicated that variation because of the position within rows or columns of the cultivation base module was relatively small compared with random error (CV =4.8% (rows) and 1.4% (columns) versus 54.2% random error).

**Figure 3:**
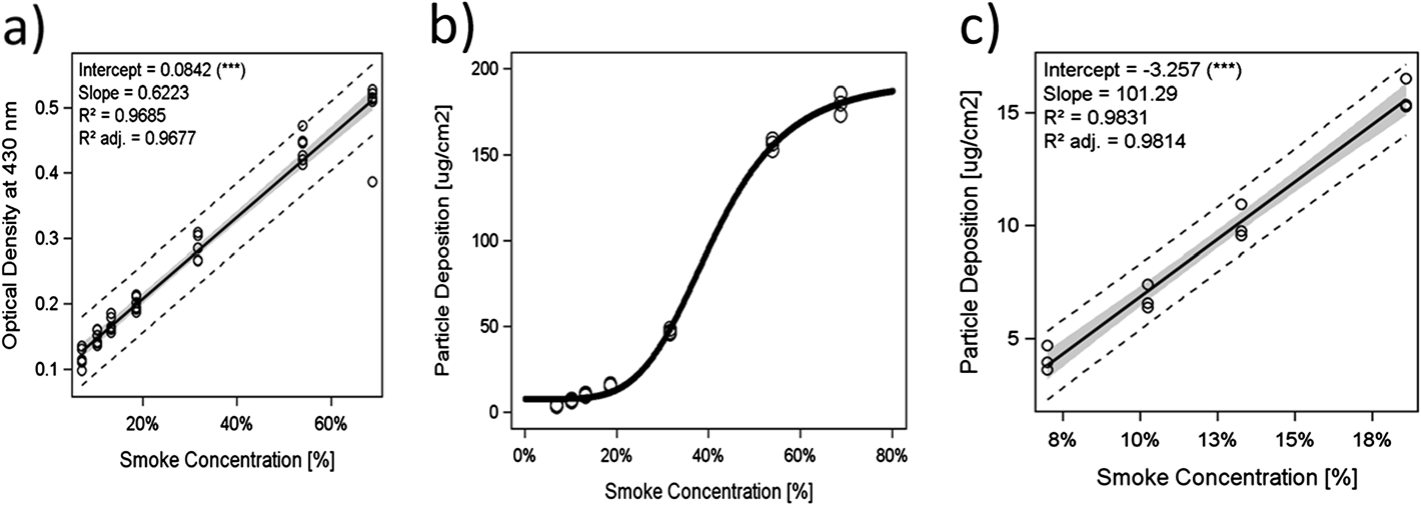
**Assessment of concentration-dependent (multi-dilution) CS exposure. (a)** The concentration-dependent change in the optical density at 430 nm upon WST-1 oxidation by CS exposure with different dilution flow rates of 0.1–3.0 L/min (7–69% CS). Data were obtained in triplicate for all dilutions. **(b)** The concentration of particles deposited on the QCM after exposure. **(c)** Linearity of particle deposition at low CS concentrations (7–19%), see insert in **(b)**.

In addition to our analysis using WST-1 as an indicator, we also applied different dilution airflows corresponding to 7–69% CS (i.e., 0.1, 0.2, 0.5, 1.0, 1.5, 2.0, and 3.0 L/min) to the Vitrocell® 24/48 system. This experiment revealed a linear correlation between the measured OD at 430 nm and the applied dilution airflow (Figure 3a).

Next, the distribution of the particulate phase of CS in the Vitrocell® 24/48 system was studied by measuring particle mass deposition on QCMs at a fixed airflow rate of 0.5 L/min (Figure 2c and d). QCMs were installed at the end of each row of the dilution/distribution system of the Vitrocell® 24/48 system. In contrast to other Vitrocell® exposure modules, installation of QCMs at specific positions on the cultivation base module is not possible with the Vitrocell® 24/48 system. Similar to what was observed for the whole CS distribution measured by WST-1 reduction, particle mass deposition on QCMs, normalized to 100%, appeared uniform over the seven rows of the dilution/distribution system of the Vitrocell® 24/48 (Figure 2c). ANOM indicated two row positions with a raw *p*-value <0.05. However, after adjustment for multiple testing (Adj. p) there was no significant difference detected between different rows of the dilution/distribution system. The mean particle mass from the three independent exposure runs was 75.7 μg/cm^2^ (Figure 2d). Variance decomposition analysis of the three exposure runs revealed that the main source of variance was again the exposure run (66.9% of total variance), leading to a CV of only 6.6% for all QCM values obtained excluding the effects from exposure runs, and 11.5% if all three runs were taken into account. The effect of the position of the QCM connected to the dilution/distribution system was a minor source of variability (8.6% of total variance). Another major source of the total variance was random error (proportionally 24.5%). In contrast to the whole CS tested in the WST-1 analysis, the slope for the deposited mass changed nonlinearly at high CS concentrations (Figure 3b). However, in the low concentration range (≤20% CS), the increase was linear (Figure 3c).

### Determination of smoke constituents inside the Vitrocell® 24/48 system

**Figure 4:**
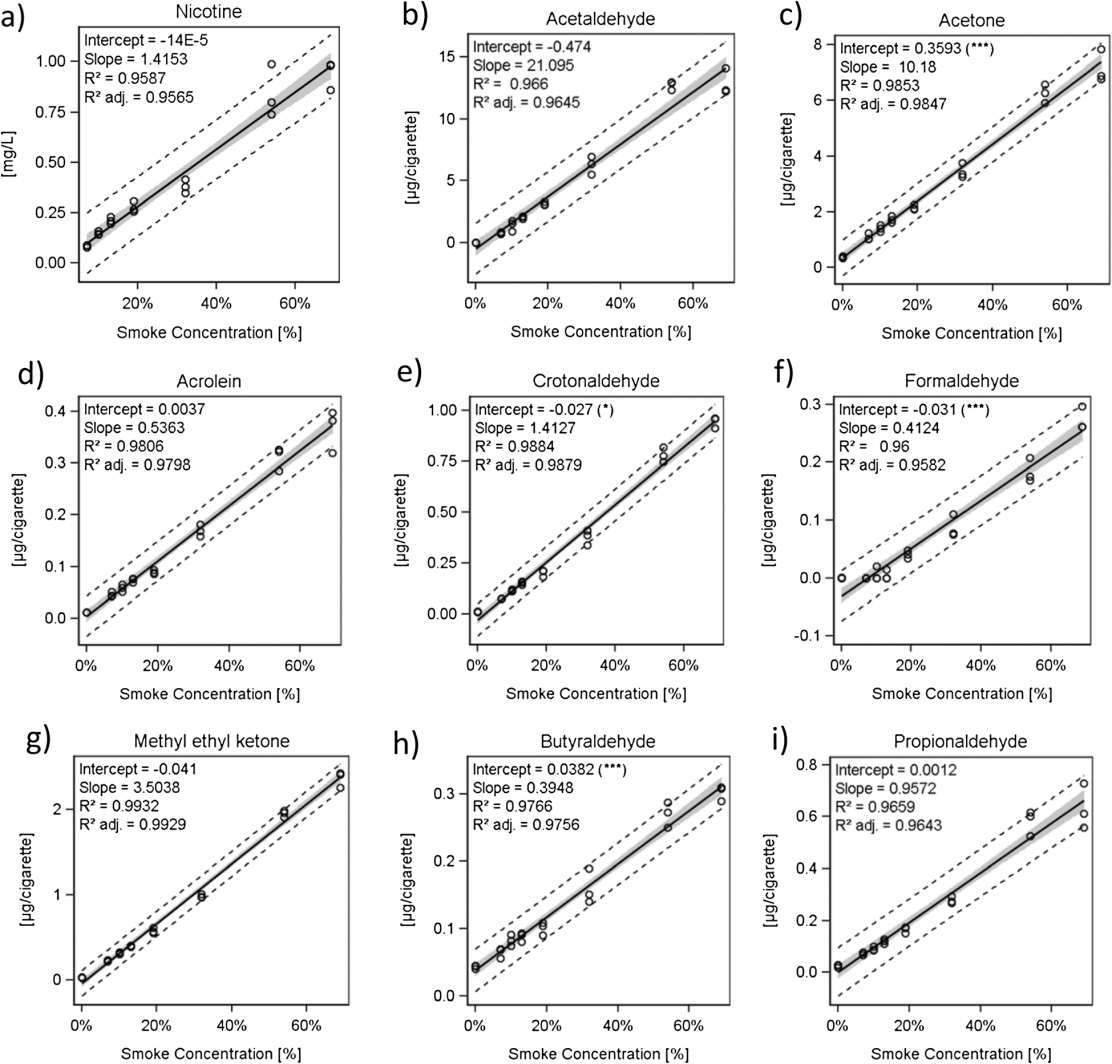
**CS application (multiple-dilution) and determination of (a) nicotine and (b)–(i) eight carbonyls inside the Vitrocell® 24/48 exposure chamber.**

To characterize the typical mainstream CS constituents delivered to the exposure system, nicotine and eight different carbonyls were trapped inside the Vitrocell® 24/48 after applying dilution airflows of 0.1, 0.2, 0.5, 1.0, 1.5, 2.0, and 3.0 L/min, followed by liquid chromatography–mass spectroscopy (LC-MS) analysis of the samples. As shown in Figure 4a, a linear correlation between trapped nicotine and CS concentration was observed (*R*^2^ > 0.95) over the entire CS concentration range (7 − 69%). Similarly, the relationships between the carbonyl concentrations, namely, acetaldehyde, acetone, acrolein, butyraldehyde, crotonaldehyde, formaldehyde, methyl ethyl ketone, and propionaldehyde, were also well-correlated with the applied CS concentration (*R*^2^ > 0.96, Figure 4b, c, d, e, f, g, h and i).

### Effect of CS on the cell viability of different cells

**Figure 5:**
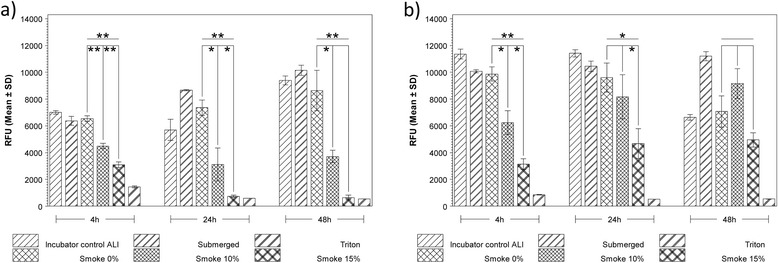
**Mainstream CS exposure of cells.** The viability based on resazurin assays of **(a)** BEAS-2B and **(b)** A549 cells following different post-exposure periods (4, 24, and 48 h). For each post-exposure time point, a different set of three inserts was used. For each treatment group (*n* =6, two smoke runs and three replicates), comparisons were performed with a mixed design ANOVA model using the exposure run as the error term. The raw *p*-values are denoted as “*” and “**” when below 0.05 and 0.01, respectively. RFU: Relative fluorescence units. Triton: Triton X-100 at 1% final concentration was used as a positive control. Incubator control ALI: non-exposed incubator controls of inserts growing at the ALI, Submerged: non-exposed cells seeded on inserts, however kept in submerged conditions. The percentages of CS used are indicated below the bar graph.

To assess the effect of CS on cells exposed in the Vitrocell® 24/48 system, the viability of A549 and BEAS-2B cells grown at the ALI were determined after exposure to fixed doses of 10% and 15% CS (dilution airflows of 1.95 and 1.25 L/min, respectively). Various post-exposure times (4, 24 and 72 h) were selected to monitor the acute effects and to account for potential recovery of cells. Figure 5a and b shows that, compared with fresh air, CS exposure rendered cells less capable of reducing resazurin, a dye commonly used to determine the metabolic activity of cells as a marker for cell viability [23]. In both cell lines, 15% CS had a consistently higher effect than 10% CS, while the effect was stronger for BEAS-2B compared to A549 cells, at 24-48 h post-exposure. 1% Triton X-100 was used as a positive control to induce a maximum decrease in viability. Overall, BEAS-2B cells were observed to be more sensitive than A549 cells to CS, with the latter showing a trend towards recovery at 24 and 48 h. As an additional control, the viability of cells seeded on membranes, however kept in submerged conditions, were similar to the incubator controls of cells lifted to the ALI.

## Discussion

In this study, we evaluated the performance of the Vitrocell® 24/48 *in vitro* exposure system for the exposure of cells to CS. The system is highly versatile because it allows simultaneous exposure of up to six replica inserts while applying multiple dilution airflows (i.e., testing seven different smoke concentrations). A total of 48 inserts can be tested with CS in a single run, or 42 test inserts and 6 control inserts exposed to fresh air. To determine whether CS was uniformly distributed across all culture inserts if a single CS concentration was applied, we used two independent methods for the whole smoke distribution and the particulate phase. WST-1 was used as a simple indicator for whole smoke, taking advantage of its sensitivity against oxidants. The mechanism and nature of the molecules in CS that are responsible for the observed reduction of WST-1 were not further investigated in this study. Superoxide is one candidate, which is present in CS and has been shown in other *in vitro* studies to be involved in the reduction of WST-1 [24]. Interestingly, for this reason, WST-1 is used also in other commercially available assays to measure the inhibition of superoxide dismutase, where superoxide is accumulating (vendors such as Sigma-Aldrich, Abcam or RnD Systems). Oxidants are present in the gas–vapor and particulate phases of smoke [21]. Thus, it was not surprising that filter trapped total particulate matter (TPM) was able to reduce WST-1 (data not shown). Whole CS applied at a single concentration to the Vitrocell® 24/48 system (0.5 L/min) reduced WST-1 in all culture inserts to a similar level, providing the first evidence that the distribution of CS was uniform in the exposure system. Furthermore, the particle mass deposition measured by QCMs attached to different rows of the dilution/distribution system was also found to be similar at the fixed CS concentration. We conducted a statistical analysis (variance decomposition) to evaluate the coefficient of variation (CV) of the Vitrocell® 24/48 system itself compared with the effect of three independent exposure runs. The CVs for different inserts containing WST-1 and for different QCMs were found to be relatively low (12.2% for WST-1 and 6.6% for QCM). With regard to CS distribution, no significant differences according to adjusted *p*-values (ANOM) could be detected in the cultivation base module (except for a single position in the WST-1 analysis, which was produced by an extreme data point in one of the three runs). Variance decomposition also allowed us to discriminate and quantify the non-random variance owing to the position (row and column) on the Vitrocell® system. Even though these estimates may be rough because of the sample size (*z*-test not significant), the row and column effect was quantitatively well below the random error, confirming equal exposure of inserts (row effects: 4.8% of total variance for WST-1 and 8.6% for QCM). Overall, the row and column variability were largely dominated by the run effect, which we believe is extraneous to the Vitrocell® 24/48 system. This may be because smoke delivery from the smoking machine is not always the same or it may originate from the assay technology, because WST-1 reduction continues post-exposure and small time differences in sample reading may contribute to run-to-run variability.

The data we collected cannot be easily compared with studies using other Vitrocell® exposure modules [13,19] because the diameters of trumpets/inserts of the exposure module, the smoking regimens, the sampling flow rates and smoking machines used were different. However, the general trends of uniform distributions and concentration-dependency are similar. For example, QCM measurements using a Vitrocell® 6/4 Stainless Steel module with a Vitrocell® VC10 Smoking Robot did not show statistical differences between different positions of the exposure module [19]. However, these researchers used a duplicate version of the same Vitrocell VC10 earlier [13] and in which study they could find statistical significance between different positions of the exposure module. This, together with the notion that the variability seen between different Vitrocell VC10’s may dependent on the service (maintenance) status of the smoking machine [25], supports our view that the most significant sources of variability are extraneous to the Vitrocell® exposure system. These studies seem to show a linear concentration-dependency of CS measured by QCM. However, they are not fully comparable in terms of the concentration range. In particular, high CS concentrations are lacking, which clearly contributed most to the non-linearity of our results using the Vitrocell® 24/48 system. It appears that, in contrast to whole smoke distribution (measured by WST-1), deposition of the particulate phase on QCMs in the Vitrocell® is governed by the airflow condition in the dilution/distribution system. In this respect, factors such as particle diffusion, impaction, and interception need to be considered to estimate the deposition rates. These can be altered by evolving aerosol due to additional mechanisms present in the flow (e.g., turbulence and electrical charging), which is a potential drawback of the Vitrocell® system. However, the deposition of particle mass at high airflow rates is still linear, which actually correspond to those flow rates we routinely use in our exposure experiments with cells. Nonetheless, the effect of different airflow rates on particle deposition should be further investigated. We believe that computational fluid dynamics (CFD) modeling will help to determine the effect of flow rate on the deposition efficiency. Our initial investigations using CFD showed that not only flow rate, but also droplet diameter, diffusion, and gravitational settling play a significant role in the process of aerosol deposition in the Vitrocell® exposure module (data not shown). In addition, *in situ* analysis of CS constituents that deposit on cells, rather than on surrogates such as the QCMs, will complement, or even improve, our understanding of the exposure levels. A different type of exposure system has been reported that provides constant flow rates while varying CS concentrations based on premixing of the smoke with air prior to the delivery to the exposure module [12]. Particle deposition in this system was reported to be linear, however it was accompanied with a considerable loss in the smoke before its delivery to the exposure module [10,11].

In this study, we also measured the effect of CS on A549 and BEAS-2B cells at the ALI. Because these cell lines resemble those of the human lung epithelium, they are frequently used as models to evaluate toxicity of the lung epithelium [26]. We found that the effects using a cell viability assay were correlated with the smoke concentration for both cell lines. BEAS-2B cells were found more sensitive than A549 cells to aerosol exposure at the ALI, which is a general phenomenon that is observed also in other toxicological studies using these cells in submerged conditions [27,28].

## Materials and methods

### Mainstream CS generation and delivery to the exposure chamber of the Vitrocell® 24/48 system

The reference cigarettes (3R4F) were obtained from the University of Kentucky (Lexington, KY, USA; www.ca.uky.edu/refcig) and were conditioned between 7 and 21 days under controlled conditions of 22 ± 1°C and relative humidity of 60 ± 3% according to ISO guidelines. 3R4F reference cigarettes were smoked according to a modified Health Canada regimen [22] (two 55 mL puffs/min, 2 s aspiration, and 8 s exhaust) on a 30-port carousel smoking machine (SM2000, Philip Morris International). To achieve continuous aerosol delivery to the Vitrocell, a total of 4 puffs/min were taken consecutively from two 3R4F cigarettes placed in the 30-port carousel (puff frequency was every 15 s). For exposures using a single CS concentration, a dilution airflow rate of 0.5 L/min was selected in the Vitrocell® 24/48 system, which corresponded to 32% of the CS.

Total Particle Matter (TPM) concentration of 100% smoke was determined by trapping undiluted cigarette smoke (3R4F) in a Cambridge-Filter, connected to the smoking machine, which yielded a concentration of 42.4 mg/L. Several dilutions of CS were used to evaluate the effect of different concentrations in the system using dilution airflows of 0.1, 0.2, 0.5, 1.0, 1.5, 2.0, and 3.0 L/min, which corresponded to %CS and TPM concentrations of 69% =29.2 mg/L; 54% =22.2 mg/L; 32% =13.0 mg/L; 19% =7.7 mg/L; 13% =5.4 mg/L, 10% =4.2 mg/L, and 7% =2.9 mg/L respectively. Fresh air exposure was used as the control. For exposures using single or multiple dilution airflows, six 3R4F reference cigarettes (66 total puffs) were smoked, giving rise to a total exposure time of 18 min. For all experiments, dilution air adjusted to 60% relative humidity was used. The temperature of the climate chamber of the Vitrocell system, as well as of the heating plate, was set to 37°C. The height of the trumpets delivering the CS to the inserts was set to 2 mm.

### Cells and culture conditions

The human alveolar basal epithelial cell line (A549; ATCC number, CCL-185) and human bronchial epithelial cell line (BEAS-2B; ATCC number, CRL-9609) were used for the exposure. Before adding the cells, 24-well polyethylene terephthalate transparent membrane inserts (ThinCert™, Greiner Bio One International AG, Kremsmünster, Austria) (pore size =0.4 μm) were preconditioned (wetted) for 4 h +/− 15 min with the cell culture medium. For culturing A549 and BEAS-2B cells, F12k (Life Technologies, Zug, Switzerland) and KGM-2 (LONZA, Basel Switzerland) media were used, respectively. Cells were thawed from the stock pool and cultured in 75 cm^2^ flasks. Confluent cell cultures were trypsinized after washing twice with phosphate buffered saline (PBS) without Ca^2+^ and Mg^2+^. The number of cells was counted using an electronic cell counter (CASY® Scharfe System, Roche Innovatis AG, Reutlingen, Germany). In brief, 50,000 A549 cells/insert and 60,000 BEAS-2B cells/insert were seeded for 24 h. Then, 18 ± 2 h before exposure, the culture medium was removed from the apical side of each insert, and cell monolayers were washed with PBS with Ca^2+^ and Mg^2+^.

### Distribution of mainstream CS in the Vitrocell® 24/48 system

The insert-to-insert variability applying a single dilution airflow (0.5 L/min) or multiple dilutions of CS was measured with WST-1 (Roche Applied Science, Rotkreuz, Switzerland). WST-1 (1 mL) was mixed with 4 mL Dulbecco’s modified Eagle’s medium supplemented with 0.1% gentamycin. A 75-μL aliquot of this mixture was filled into the cell culture inserts and exposed to six 3R4F reference cigarettes (66 total puffs). The total exposure time was 18 min. After exposure to CS, 50 μL WST-1 mixture was transferred into a 96-well plate, and the optical density was measured at 430 nm. To determine the effect of different runs on the overall variability, OD measurements from three independent smoke exposure runs were analyzed.

In the same CS exposure runs in which the WST-1 colorimetric assay was applied, particle deposition was monitored by connecting individual Vitrocell® quartz crystal microbalances (Vitrocell® Systems GmbH, Waldkirch, Germany) to each row of the dilution/distribution system. During exposure, deposition of particles was monitored online, and the final mass after the last cigarette was smoked was taken to compare the particle distributions with different CS concentrations.

### Viability assay

Cell inserts (*n* = 3) were exposed in two independent exposure runs with six cigarettes of 3R4F with 15% and 10% mainstream smoke inside the Vitrocell®. The total exposure time was 18 min. The cell viability was measured with a resazurin assay at 4, 24, and 48 h post-exposure. After the post-exposure periods, 150 μL resazurin diluted in culture medium (120 μg/mL) (Sigma-Aldrich, Buchs, Switzerland) was added per insert and incubated for 2 h at 37°C with 5% CO_2_ and 95% relative humidity. At the end of the incubation time, samples of 100 μL/insert were removed, and the fluorescence was measured for each well using 560 nm excitation and 590 nm emission wavelengths. A final concentration of 1% Triton has been added to the basal media of ALI inserts, as a positive control to induce maximum loss in cell viability. Non-exposed incubator controls of inserts growing at the ALI were used as negative control. Non-exposed cells seeded on inserts, however kept in submerged conditions, were used as an additional control.

### Determination of eight carbonyls by liquid chromatography–electrospray ionization–tandem mass spectrometry

Carbonyl compounds were trapped during CS smoke exposure in the Vitrocell® 24/48 system by filling the rows of the cultivation base module with 18.5 mL PBS solution per row. Ten 3R4F cigarettes were smoked for each exposure run. A total of three independent exposure runs were conducted, containing technical triplicates. Multiple dilutions of CS smoke were performed to determine the carbonyl concentration at dilution airflows of 0.1, 0.2, 0.5, 1.0, 1.5, 2.0, and 3.0 L/min. As a blank, we also collected samples from fresh air exposed PBS. The total exposure time was 28 min. After the exposure was completed, an aliquot of 1.2 mL exposed PBS was incubated with 1.8 mL dinitrophenylhydrazine (DNPH) solution (15 mM DNPH in acetonitrile and 25 mM perchloric acid) for 30 min at room temperature, and chemical derivatization was quenched by addition of 150 μL pyridine. A 500 μL aerosol-derivatized sample was introduced in a LC-MS glass vial previously filled with 485 μL acetonitrile and 15 μL internal standard working solution containing acetone-*d*_*6*_-DNPH and methyl-ethyl-ketone-*d*_*5*_-DNPH (24 μg/mL, each).

Formaldehyde–DNPH, acetaldehyde–DNPH, acetone–DNPH, crotonaldehyde–DNPH, propionaldehyde–DNPH, acrolein–DNPH, methyl-ethyl-ketone–DNPH, and butyraldehyde-DNPH were analyzed by liquid chromatography (Agilent 1200) coupled to electrospray ionization tandem mass spectrometry (5500 QqQ, AB Sciex). Separation of the aldehyde was performed in isocratic mode on a chromolith speedrod RP-18e HPLC column using water, acetonitrile, tetrahydrofuran, and isopropanol (59:30:10:1, v/v/v/v) at a flow rate of 2.5 mL/min (column set at 40°C equipped with a post-column splitter 1:6 before entrance into the MS). MS detection was realized in multiple reaction monitoring mode, and the carbonyl compound concentration (expressed in μg/cigarette) was calculated using an external calibration curve.

### Determination of nicotine

For the determination of the nicotine concentration in diluted CS, smoke was collected using Extrelut 3NT columns (Merck, Zug, Switzerland) impregnated with 2 mL of a 0.5 M H_2_SO_4_ solution. The analyzed smoke concentrations were 69%, 54%, 32%, 19%, 13%, 10%, and 7%. The Extrelut 3NT tubes were connected at the exhaust of the dilution system of the Vitrocell® 24/48 system. For each smoke concentration, three samples were analyzed and a total of three independent exposure runs were conducted. After sample collection, the internal standard isoquinoline was added to the top of the column, and the trapped nicotine including the internal standard was eluted with a mixture of 5 vol% triethylamine in *N*-butylacetate. In brief, 1 μL of the extract was injected into an Agilent 7890A gas chromatograph (Santa Clara, CA, USA)in split mode using a split ratio of 1:20 and injector temperature of 220°C. The separation was performed at an oven temperature of 140°C in isothermal mode using a 15 m DB-5 fused silica capillary column with an internal diameter of 0.25 mm and 0.25 μm film thickness. Helium was used as carrier gas, and the column flow was set to 1.4 mL/min. Compounds were detected using flame ionization detection, and nicotine was quantified according to an internal standard calibration curve.

### Statistical analysis

Variance decomposition was performed with SAS 9.2 using the procedure MIXED and the COVTEST option and the restricted maximum likelihood method. Effects such as row, column, and exposure run were added as random effects in the model. For an optical density of 430 nm, the mean value per exposure run for fresh air was subtracted from all individual measurements. The *p*-values refer to a Wald *z*-test, which examines if the variance of a random effect is significantly different from zero. ANOM was applied to detect significance between WST-1 reduction of different insert positions or between different QCMs. The raw *p*-values were adjusted for multiple testing according to Nelson-Hsu. The statistics for the cell viability assays was based on mixed design ANOVA model.

## Conclusions

Our characterization of the Vitrocell® 24/48 system demonstrates that the system is well-suited for CS exposure of cells growing at the ALI, based on its overall performance towards the distribution of smoke to inserts of the exposure module. The system did not reveal a major technical bias for low CS concentrations, up to 20%, which however correspond to a concentration range high enough to cause a decrease in cell viability in two different cell lines tested. This range was also sufficient to induce alterations in smoking-related biological networks, verified by results obtained from recent CS exposure experiments we conducted on 3D-organotypic epithelial cells using the Vitrocell® 24/48 system [7-9]. Our characterization also revealed that aspects such as particle mass deposition rates in the Vitrocell® 24/48 system need to be further investigated, in order to judge on dose-effects at higher CS concentrations. We believe the WST-1 methodology we used in this study adds a simple tool to measure whole smoke exposure of inserts, beyond particle mass deposition measurements. However more specific tools are still lacking and therefore we have limited understanding concerning the gas–vapor phase depositing on cell inserts. Analyzing metabolites from CS constituents after exposure of cells maybe one way to address this in future.

## Abbreviations

ATCC: American type culture collection
ALI: Air–liquid interface
DNPH: Dinitrophenylhydrazine
PBS: Phosphate buffered saline
QCM: Quartz crystal microbalance
OD: Optical density
MS: Mass spectroscopy
CS: Cigarette smoke
HPLC: High-performance liquid chromatography
TPM: Total particulate matter
